# The Anatomy of Suicide

**Published:** 1841-07

**Authors:** 


### ART. IX.

*The Anatomy of Suicide.* By FORBES WINSLOW, Member of the Royal College of Surgeons, &c.—*London*, 1840. 8vo, pp. 339.

HAD this work not been rather of a popular than of a professional character, we would have noticed it sooner. The variety of anecdotes which it contains may render it in some degree attractive to the general reader, but there is not much to interest the philosopher or the physician. We will notice briefly the several heads under which Mr. Winslow distributes his subject.

Chap. i. is on the "suicides of the ancients." It is written in a sufficiently entertaining manner, and contains divers instances of suicide among the ancients, and some notice of the opinions and laws of the Greeks and Romans regarding it; but here, as in other parts of the work, the author is much too easy as to the evidence on which his cases are adduced. For example (p. 17) he tell us, with great gravity, that when Empedocles threw himself into the crater of Mount Etna, his fate was not discovered till one of his sandals was ejected from the volcano. Setting aside the conceit of the sandal, Mr. Winslow ought to have known that the story about Empedocles and Mount Etna rests on no sort of testimony, and that the real manner of the philosopher's death is quite uncertain.†

In chap. ii. our author notices and confutes "various writers in defence

† Diogenes Laertius, de vitis Philosophorum, in vitâ Empedoclis.

of suicide," and expresses himself with no great amenity towards David Hume, calling him "the detestable author of this abominable treatise," &c. We dislike many of Hume's opinions just as much as Mr. Winslow can do, and think that his metaphysical powers have been over-rated; but we are not on that account disposed to deny that he was a philosophic, candid, and amiable man. We cannot think it much worth while to argue with the advocates of a crime, from which reason, religion, and instinct alike recoil. Mr. Winslow, however, in chap. iii. exerts himself to prove that suicide is "a crime against God and man," wherein we shall content ourselves with entirely acquiescing. He also argues that suicide is an act of cowardice, not courage, a point which admits of more discussion. Any difficulty which the question may appear to present arises, we think, from not discriminating sufficiently between physical and moral courage. We regard suicide as an act of moral cowardice, and we believe that the degree of physical courage of the individual has very little to do with the matter; for it happens in many other instances besides that of suicide, that an utter subversion of all moral firmness and self-possession causes a suspension of the instinct of self-preservation. This has often been exemplified in the valour of men, and of women too, who have fought desperately because they were desperately frightened; the general sense of fear so overwhelming the moral and intelligent being, that the actual physical causes of danger are made light of, and rashly encountered. An amusing illustration of this is contained in James Hogg's tale of "Basil Lee." The hero performs prodigies of valour, and gets the reputation of being the bravest man in the British army because, at the approach of battle, he is so transported with terror that he has only one idea left, which is a vague though intense conception that everything is to be exterminated; and accordingly, he lays about him with wonderful energy and effect. We believe that suicide might be committed by the bravest man in the world, as well as by the man most deficient in personal courage; but no man of moral courage would commit an act which implies an utter loss of all self-dependence, as well as all dependence upon Providence.

Chap. iv. is "on the influence of certain mental states in inducing the disposition to suicide." There is here a good deal of interesting matter, and a number of cases, some of which would have been more valuable if the source whence they were derived had been stated. Some of the instances, also, are irrelevant. Thus, at p. 74, we are told that the late Mr. Mathews, the comedian, knocked a very little man off a very tall horse, in obedience to a sudden and irresistible impulse, arising from an over-excited state of the brain: and at p. 75, we learn the fate of some teacups which fell victims to a blind propensity to destroy, manifested by a woman in the Asylum at Wakefield. We do not mean to say that such cases have no connexion with the subject of suicide, but that the author has omitted to point out the connexion, and placed them heterogeneously among his instances of suicide from a blind impulse.

In a note at the bottom of p. 101 we are somewhat surprised to find the following words:

"A phenomenon attends the dying moment which we do not recollect to have seen noticed. A man who fell into the water, and who rose several times to the surface, had a consciousness of the hopelessness and awfulness of his situation; he felt that death was inevitable. With this conviction on his mind he saw presented to him a picture of his past life; the minutest action in which he had been engaged was brought in a kind of tableau before him. Circumstances which had long been forgotten were conjured up from his brain, and he had a bird's eye view of his past career. Possibly this may occur to every person at the moment of dying. The expressions of those placed under such circumstances would indicate as much."

We must observe that the coincidence between the above case and one mentioned in the Confessions of an English Opium Eater is rather singular, and that the generalization of the fact (supposing it to be one) is rather hasty. We cannot here enter into so metaphysical a subject, but we may, in passing, express a doubt whether, under any circumstances of life or death, a created mind could take in the survey of a lifetime in a moment; because this would imply an independence of *time* and of the *succession of ideas*—a condition which we apprehend is peculiar to the Deity.

Chap. v., on "imitative or epidemic suicide," is a short one; and the author has perhaps done well not to enter into the philosophy of a subject which involves some of the most obscure points both of physiology and of morals.

Chap. vi., on "suicide from fascination," is also brief. The conclusion is amusing. In reference to the disposition to suicide by precipitation from a height, the author observes that "persons who are subject to feelings of this character should be advised to avoid ascending elevated places."

Chap. vii. is on "the enthusiasm and mental irritability which, if encouraged, would lead to suicide." The morbid excitability frequently attendant on high development of the mental powers is here illustrated by several anecdotes of distinguished men. Speaking of this with reference to Byron, the author observes, "There can be no doubt that it was but the natural effect of a peculiar condition of nervous function; but instead of endeavouring to subdue the feeling, *he did his best to encourage it.*" The last words, we believe, contain the key to many of the peculiarities of men of genius. The truth is, that men of genius, especially in the imitative arts, are too frequently men of vanity; they love to attract attention, and develope various modes of "excitability," precisely on the same principle that some young ladies scream and faint.

Chap. viii. is on the "physical causes of suicide." These are referred to climate, season, cerebral diseases and injuries, physical suffering, diseases of the digestive organs complicated with melancholia and hypochondriasis, suppressed secretions, various forms of vice and intemperance, insanity, and hereditary disposition. In regard to the effects of season, Mr. Winslow combats the prevailing notion that November is the month in which most suicides are committed, and contends that "in all the European capitals when anything approaching to correct statistical evidence can be procured, the *maximum* of suicide is in the months of June and July; the *minimum* in October and November." (pp. 131-2.) With respect to disease as a predisposing cause of suicide, he insists much on the frequency of chronic disease of the brain, arising from injury or other causes, and cites Dr. Mantell's opinion as to the influence of slight but neglected injuries of the head in causing a disposition to self-destruction.

In reasoning on the influence of abdominal disease in inducing hypochondriasis and melancholy, Mr. Winslow makes the singular observation, "that all impressions arising from the viscera of the abdomen are naturally obscure." (p. 148.) We take leave to doubt this. Are the sensations of hunger, thirst, and nausea obscure sensations, or are the pains of cramp in the stomach and colic obscure pains?

Chapters ix. and x. are on "the moral and physical treatment of the suicidal disposition." They are perhaps the best in the book. Mr. Winslow very properly inculcates the expediency of removing a patient who shows a disposition to self-destruction from the scenes and associations to which he has been accustomed. He dwells, with equal truth, on the benefits which arise from cultivating a love of nature and an interest in the affairs, and a sympathy in the misfortunes of our fellow-beings. The contemplative frame of mind engendered by the intense feeling of natural beauty is often mistaken by the vulgar for melancholy; but we believe it to be in reality one of the surest preservatives against that state. We never knew a genuine disciple of Wordsworth who was a melancholy man. Again, it may seem paradoxical to maintain that an active participation in the misfortunes of others can win us from our own sorrows, and restore cheerfulness to the desponding mind. Yet such is the fact. We gain wisdom and strength by comparing ourselves with others, and our destiny with theirs. We find them bearing up against the very evils that we are sinking under; we see them sinking under evils which to us appear trivial: thus strength springs even from reciprocal weakness, and endurance from the interchange of affliction. Grief is indolent, benevolence is active; and in our successful exertions to overcome the difficulties or assuage the miseries of others, we often discover how much we have been wanting to ourselves, and find an apparently overwhelming evil resolved into our own want of fortitude and activity.

In chapter xi. our author argues the question whether suicide be the result of insanity? His general answer is in the affirmative, and we think he is right. It appears to us that there are two widely different states which singly or combined may conduce to suicide. The one is a state of *perverted instinct*, in which a blind propensity to self-destruction supersedes the instinct of self-preservation: a state parallel to that in which a mother destroys her child without being able to assign any cause for it but an irresistible impulse. The other is a state of moral depression, caused by the consciousness of evils which are either in themselves of dreadful magnitude, or which the mind of the individual is too feeble to bear. In this state the sufferer, though he still fears death, and perhaps trembles at the unknown futurity into which he is about to plunge, still dreads nothing so much as his present anguish, and thinking that any change must be for the better, voluntarily puts a period to his earthly existence.

Now it will not be disputed that the first of these states, that of perversion of the most powerful instinct of nature, constitutes a form of mania. With respect to the second state, it should be remembered that the evil which is thought intolerable, and to which death itself is preferred, is usually one which a vigorous and well-poised mind would soon shake off; and that very few evils are insupportable if viewed in a just light and met in a proper spirit: the very disposition, therefore, to regard any of the ordinary evils of life as utterly unendurable implies either a perversion of ideas as to the fact, or a very enfeebled condition of the moral powers, either of which is sufficient to constitute melancholy. When to these considerations we add, what is truly stated by our author, that in a great majority of cases a careful enquiry into the previous conduct of the suicide would afford strong indications of insanity, we think we are justified in assuming the general position that suicide is the result of madness. We entirely assent also to the opinion of Mr. Winslow, expressed in another chapter, that in the few cases which may be doubtful, the unhappy individual should have the benefit of the doubt, and that the verdict *felo de se* should never be returned.

Chap. xii. treats of "suicide in connexion with medical jurisprudence." It contains most of the information to be found in the books on legal medicine; the subject of suicide by poisoning is, however, scarcely alluded to.

Chap. xiii. is on the "statistics of suicide." It contains much information and numerous tables, derived from the reports of the Statistical Society of London, the work of M. Guerry on the Moral Statistics of France, the paper by M. Prevost in the Bibliothèque Universelle, on Suicide in the Canton of Geneva, and other sources. These documents being well known we need not here attempt any condensation of their contents, which is the less needful with respect to the memoir of M. Prevost, inasmuch as an analysis of it has been given in the second volume of this journal, from which Mr. Winslow has copied his statement* verbatim without acknowledgment. He does, however, express in his preface a general obligation to certain books, and to this journal among the rest.

The two remaining chapters, one treating of the appearances presented after death in the bodies of suicides, and the other containing a collection of singular cases, do not call for any special notice.

Considered as a literary production Mr. Winslow's book is not of an elevated order. The style is diffuse and often incorrect. Many passages are feeble both in thought and expression, and some are altogether devoid of meaning. Thus at p. 64 the author talks of a young gentleman "of high natural and acquired attainments." In these few words we have a *bull* and a pleonasm: how can an *attainment* be *natural?* and if not, how can it be otherwise than *acquired?* At p. 334, he says, "the end of punishment is to prevent the criminal from doing further injury, as well as to *induce* others *from* committing similar offences." And so of many other passages. The general reader will, however, we dare say, pronounce a more favorable judgment on the Anatomy of Suicide; which is certainly more deserving of perusal than Mr. Winslow's former work, entitled Physic and Physicians, the blunders of which were inconceivable.

* Vide British and Foreign Med. Rev., vol. II., p. 277.

